# Draft Genome Sequence of FGL03-6, a Race 1 Strain of Fusarium oxysporum f. sp. conglutinans, the Causal Agent of Cabbage Fusarium Wilt

**DOI:** 10.1128/genomeA.00191-18

**Published:** 2018-04-19

**Authors:** Honghao Lv, Yuhong Yang, Xing Liu, Jian Ling, Zhiyuan Fang, Limei Yang, Yangyong Zhang, Yong Wang

**Affiliations:** a Institute of Vegetables and Flowers, Chinese Academy of Agricultural Sciences; Key Laboratory of Biology and Genetic Improvement of Horticultural Crops, Ministry of Agriculture, Beijing, China

## Abstract

We present here the draft genome sequence of FGL03-6, a race 1 strain of Fusarium oxysporum f. sp. conglutinans, the pathogen causing Fusarium yellows of cabbage. The FGL03-6 genome consists of 414 scaffolds with 61,662,789 bp (GC content, 47.9%) and 9,790 predicted genes.

## GENOME ANNOUNCEMENT

Fusarium oxysporum is a soilborne fungus that includes both pathogenic and nonpathogenic strains. The pathogenic strains can infect more than 120 different plant species, including more than 100 economically important crops (1). The fungus invades from the host roots and colonizes in the xylem tissues to cause yellowing or wilting disease. Due to its broad host range, F. oxysporum can be divided into more than 120 formae speciales, some of which can be further divided into several physiological races (2). Some formae speciales are ideal models to use for studying plant-fungi interactions (3).

Cabbage Fusarium wilt (CFW) disease is a worldwide threat for cabbage production, resulting in severe production loss of almost all cole crops. The causal agent of CFW is Fusarium oxysporum f. sp. conglutinans. CFW was first observed by Smith in the United States in 1895 (4) and has now spread almost all over the world. F. oxysporum f. sp. conglutinans populations with high pathogenicity have been emerging in China in recent years (5).

So far, only two races for F. oxysporum f. sp. conglutinans have been discovered (6, 7). Here, we report the draft genome sequence of a highly virulent race 1 strain, FGL03-6, which was isolated in 2008 from diseased cabbage plants in Yanqing, Beijing, China (8, 9). The fungus was cultivated on potato dextrose agar (PDA) medium, and 500 mg of mycelium was used for DNA extraction with the DNeasy plant minikit (Qiagen, Inc., Shanghai, China). Approximately 50 μg genomic DNA was subjected to whole-genome resequencing using the HiSeq X Ten platform (Illumina, Inc., San Diego, CA, USA).

To obtain a high-quality genome sequence, three libraries were constructed, specifying 150-bp paired-end reads with insert sizes of 500, 2,000, and 6,000 bp. In total, 5.08, 2.52, and 2.588 Gb of raw data were received, respectively. The genome was assembled using the programs SOAP*denovo* 2.04 and GapCloser 1.12 (10). Assembly quality was assessed by examining the *N*_50_ value and by examining the total number of scaffolds produced by the programs. Scaffolds smaller than 500 bp were removed through a Perl script. The protein-coding genes were predicted from the assembly, using AUGUSTUS version 3.2.2 (11), based on gene models from Fusarium oxysporum. The assembly was 61,662,789 bp (GC content, 47.9%) in length, with an *N*_50_ value of 1,209,780 bp, resulting in 414 scaffolds. There were 9,790 predicted coding genes in total, with an average length of 1,313 bp.

The genomic data of the highly virulent F. oxysporum f. sp. conglutinans strain FGL03-6 will contribute to our knowledge of F. oxysporum f. sp. conglutinans genomic diversity and provide evidence for F. oxysporum f. sp. conglutinans-host interaction and for prevention of this disease.

### Accession number(s).

This whole-genome shotgun project has been deposited at DDBJ/ENA/GenBank under the accession no. NRIA00000000. The version described in this paper is version NRIA02000000.
